# “Iron” road in apple: the pathway regulated by MPK6-2–ERF4–FIT module

**DOI:** 10.1093/plphys/kiaf604

**Published:** 2025-11-19

**Authors:** Munkhtsetseg Tsednee

**Affiliations:** Assistant Features Editor, Plant Physiology, American Society of Plant Biologists; Research Center for Environmental Changes, Academia Sinica, Taipei 11529, Taiwan

Plants need iron (Fe) nutrient for their photosynthesis and respiration. Despite its high abundance in the earth's crust, Fe is bio-unavailable for plant uptake in alkaline soil, which accounts for about one-third of the world's arable land (Riaz and Guerinot 2021). This limited availability leads to Fe deficiency in plants and becomes a limiting factor for crop yield and quality (Briat et al. 2015). While Fe regulatory mechanisms governing its acquisition from calcareous soil have been well documented in model plants, they remain unexplored in fruit trees.

In a recent issue of *Plant Physiology*, Wu et al. (2025) investigated the Fe deficiency response in apple (*Malus xiaojinensis*) and identified a regulatory pathway, mediated by the MxMPK6-2 (Mitogen-activated protein kinase 6-2)-MxERF4 (Ethylene response factor 4)-MxFIT (FER-like iron deficiency-induced transcription factor) module, for activating its Fe uptake.

Previously, MbERF4 was shown to suppress rhizosphere H^+^ secretion in apple rootstock *M. baccata* under Fe-limited conditions (Zhang et al. 2020). Building on this, Wu et al. (2025) used their transgenic apple lines overexpressing (OE-*MxERF4*) and silencing (RNAi-*MxERF4*) the *MxERF4* to investigate its role in low Fe. OE-*MxERF4* plants displayed exacerbated Fe-deficiency symptoms, including reduced chlorophyll, lower Fe contents, and downregulation of Fe-uptake related genes *MxIRT1* (*Iron transporter 1*) and *MxFRO2* (*Ferric reductase oxidase 2*) (Riaz and Guerinot (2021). In contrast, RNAi-*MxERF4* lines showed enhanced tolerance to Fe deficiency, suggesting that MxERF4 functions as a negative regulator in Fe deficiency response.

Although *MxERF4* transcript accumulated under Fe limitation, its protein level declined, prompting the authors to explore its post-translational regulation. Through yeast 2-hybrid screening and subsequent in vitro and in vivo interaction assays, they identified MxMPK6-2 as an interacting kinase that phosphorylates MxERF4 at Ser14 and Thr107. Indeed, this phosphorylation promotes MxERF4 protein degradation, as demonstrated by cell-free degradation assays showing accelerated and slowed turnover of MxERF4 in OE-*MxMPK6-2* and RNAi-*MxMPK6-2* apple calli, respectively. Consistently, OE-*MxMPK6-2* calli also exhibited a rapid reduction in MxERF4 protein levels under Fe deficiency (Wu et al. 2025).

As expected, phosphorylation of MxERF4 in planta alleviates its negative regulatory effect on Fe deficiency response, as seen with elevated Fe content and enhanced Fe-uptake activity in OE-*MxMPK6-2* roots (Wu et al. 2025). Since MxMPK6-2 was previously reported to promote Fe acquisition in apple rootstock by positively regulating root acidification (Sun et al. 2023), it is likely that this kinase plays a critical role in coordinating Fe deficiency responses in apple.

Furthermore, to elucidate the regulatory mechanism of MxERF4, Wu et al. (2025) employed yeast 2-hybrid assays to identify its potential interacting partners among key transcriptional regulators, including MxFIT and bHLH transcription factors involved in Fe homeostasis (Zhang et al. 2023). Using complementary in vitro and in vivo assays, they confirmed a direct interaction between MxERF4 and MxFIT.

In model plants, FIT forms heterodimers with bHLH transcription factors, such as bHLH38, bHLH39, and bHLH100, to orchestrate Fe-deficiency responses (Schwarz and Bauer 2020). Through in vivo interaction and DNA-binding assays, the authors verified that MxFIT retains this conserved function in apple by forming heterodimers with MxbHLH38/29 transcription factors to activate downstream Fe-uptake genes. Notably, MxERF4 binding to MxFIT interferes with MxFIT-MxbHLH38/39 heterodimer formation, based on yeast 3-hybrid and split-luciferase complementation assays. Promoter-reporter analysis further revealed reduced transcriptional activation of *MxIRT1* and *MxFRO2* when MxFIT and MxbHLH39 were coexpressed with MxERF4 (Wu et al. 2025). This indicated that MxERF4 disrupts heterodimer formation, thereby attenuating transcriptional activation of Fe uptake–related genes.

Lastly, using wild-type MxERF4 and its phosphorylation site mutants (MxERF4^S14D/T107D^), Wu et al. (2025) demonstrated that the phosphorylation of MxERF4 weakens its interactions with MxFIT and restores MxFIT-MxbHLH38/39 heterodimer formation. Phospho-mimic transgenic lines, showing enhanced tolerance to Fe deficiency, also confirmed the functional relevance of this regulatory mechanism in planta.

In summary, under Fe-deficient conditions, MxMPK6-2 phosphorylates MxERF4, leading to its degradation and reducing its interaction with MxFIT. The consequent release of MxFIT enables its heterodimer formation with MxbHLH38/39, thereby activating the Fe uptake genes *MxIRT1* and *MxFRO2* (Fig.). These findings uncover the molecular basis underlying Fe uptake regulation in apple and highlight potential genetic targets for the development of Fe-efficient apple rootstocks.

**Figure.**
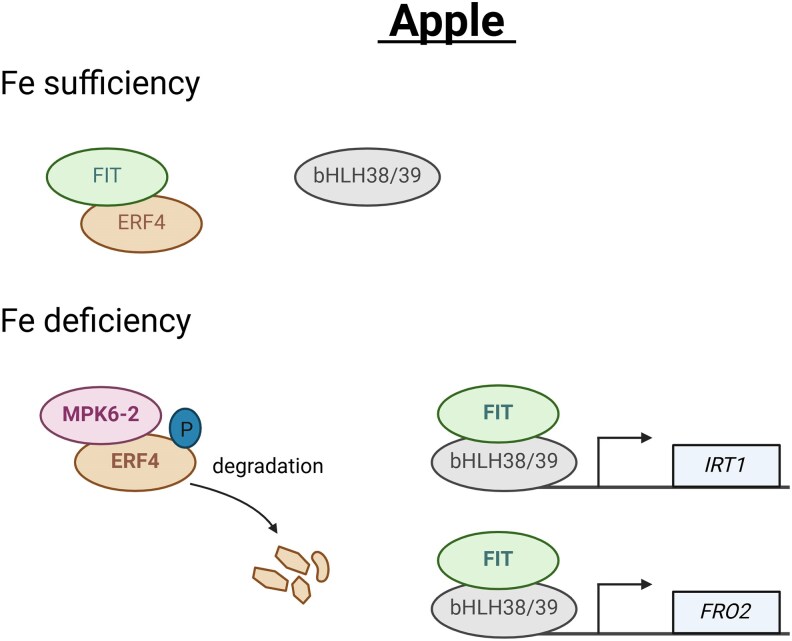
Proposed model of the MPK6-2–ERF4–FIT signaling module in regulating Fe deficiency responses in apple rootstocks. Under Fe sufficiency, MxFIT is inactive and unavailable to form heterodimers with MxbHLH38/29. Under Fe deficiency, MxMPK6-2 phosphorylates MxERF4, promoting its degradation and reducing its interaction with MxFIT. This releases MxFIT to form heterodimers with MxbHLH38/39, thereby activating transcription of the Fe uptake genes *MxIRT1* and *MxFRO2* (modified from Wu et al. (2025)).

## Data Availability

No new data were generated or analyzed for this article.

## References

[kiaf604-B1] Briat JF, Dubos C, Gaymard F. Iron nutrition, biomass production, and plant product quality. Trends Plant Sci. 2015:20(1):33–40. 10.1016/j.tplants.2014.07.005 25153038

[kiaf604-B2] Riaz N, Guerinot ML. All together now: regulation of the iron deficiency response. J Exp Bot. 2021:72(6):2045–2055. 10.1093/jxb/erab003 33449088 PMC7966950

[kiaf604-B3] Schwarz B, Bauer P. FIT, a regulatory hub for iron deficiency and stress signaling in roots, and FIT-dependent and -independent gene signatures. J Exp Bot. 2020:71(5):1694–1705. 10.1093/jxb/eraa012 31922570 PMC7067300

[kiaf604-B4] Sun QR, Zhao DR, Gao M, Wu Y, Zhai LM, Sun S, Wu T, Zhang XZ, Xu XF, Han ZH, et al MxMPK6-2-mediated phosphorylation enhances the response of apple rootstocks to fe deficiency by activating PM H-ATPase MxHA2. Plant J. 2023:116(1):69–86. 10.1111/tpj.16360 37340905

[kiaf604-B5] Wu Y, Sun Q, Zhao D, Zhang X, Zhai L, Lv J, Wu T, Zhang X, Han Z, Wang Y. MPK6-2-mediated phosphorylation of ERF4 releases FIT and enhances Fe uptake in apple. Plant Physiol. 2025. 10.1093/plphys/kiaf657 41413893

[kiaf604-B6] Zhang GF, Liu W, Feng Y, Li DY, Li KT, Sun QR, Zhai LM, Wu T, Zhang XZ, Xu XF, et al Ethylene response factors MbERF4 and MbERF72 suppress iron uptake in woody apple plants by modulating rhizosphere pH. Plant Cell Physiol. 2020:61(4):699–711. 10.1093/pcp/pcz234 31868909

[kiaf604-B7] Zhang ZX, Cheng J, Wang WX, Gao YL, Xian XL, Li CL, Wang YX. Transcription factors dealing with iron-deficiency stress in plants: focus on the bHLH transcription factor family. Physiol Plant. 2023:175(6):e14091. 10.1111/ppl.14091 38148182

